# Baseline OCT biomarkers associated with visual prognosis in cystoid macular edema secondary to noninfectious uveitis

**DOI:** 10.1007/s00417-026-07292-3

**Published:** 2026-05-25

**Authors:** Muhiddin Fatih Bodur, Nurullah Koçak, Hilal Eser-Ozturk, Yuksel Sullu

**Affiliations:** 1 Department of Ophthalmology, Samsun City Hospital, Canik/Samsun, 55080 Turkey; 2 Department of Ophthalmology, Ondokuz Mayis University, Samsun, Turkey

**Keywords:** Cystoid macular edema, Uveitis, Optical coherence tomography, Ellipsoid zone, Disorganization of retinal inner layers, Subretinal fluid, Visual prognosis

## Abstract

**Purpose:**

To evaluate the relationship between baseline optical coherence tomography (OCT) biomarkers and both baseline and post-treatment best-corrected visual acuity (BCVA) in eyes with cystoid macular edema (CME) secondary to noninfectious uveitis.

**Methods:**

This retrospective study included 84 eyes from 66 patients with uveitic CME showing anatomical resolution after treatment. Baseline OCT parameters, including central foveal thickness (CFT), subretinal fluid (SRF) height, total intraretinal cyst (IRC) volume, hyperreflective foci (HRF) count, and integrity of the ellipsoid zone (EZ), external limiting membrane (ELM), and disorganization of retinal inner layers (DRIL), were analyzed. Univariate and multivariate linear regression analyses were used to identify predictors of baseline and final BCVA.

**Results:**

In univariate analysis, baseline BCVA was significantly associated with CFT (*p* < 0.001), DRIL presence (*p* = 0.021), and SRF height (*p* = 0.002). In multivariate analysis, CFT (*p* = 0.038) and SRF height (*p* = 0.049) remained independent predictors. For final BCVA, univariate associations were found with EZ (*p* = 0.007) and ELM disruption (*p* = 0.007), total IRC volume (*p* = 0.010), and baseline CFT (*p* < 0.001), whereas only baseline CFT (*p* = 0.006) persisted as an independent predictor.

**Conclusion:**

Increased baseline CFT was significantly associated with poorer visual acuity both at baseline and after treatment, emerging as the strongest independent predictor of visual outcome. In univariate analysis, SRF height, EZ and ELM disruption, DRIL presence, and higher total IRC volume showed associations with BCVA; however, these relationships were not retained in the multivariate model and therefore were not independent predictors after adjusting for covariates.

## Abstract (highlights)

**Key messages:**

**What is known:**

OCT biomarkers such as central foveal thickness, outer retinal integrity, and disorganization of retinal inner layers are associated with visual function in uveitic cystoid macular edema.

Anatomical improvement, reflected by a reduction in central macular thickness, does not always parallel functional visual recovery, making visual prognosis clinically challenging to predict.

**What is new:**

Increased baseline central foveal thickness was the strongest and only independent predictor of post-treatment visual acuity in eyes with noninfectious uveitic cystoid macular edema.

Although DRIL, subretinal fluid height, ellipsoid zone and external limiting membrane disruption, and intraretinal cyst volume were associated with visual acuity in univariate analyses, they were not independent predictors after multivariate adjustment.

Baseline central foveal thickness may serve as a simple and practical OCT-based prognostic marker for visual outcomes in routine clinical assessment of uveitic macular edema.

## Introduction

Cystoid macular edema (CME) secondary to uveitis is one of the most important and potentially reversible causes of visual impairment in these patients [1]. Optical coherence tomography (OCT) plays a central role in clinical monitoring by providing detailed structural information that reflects disease activity and treatment response [2]. However, in uveitic CME, the structure–function relationship is not always strong; improvement in central thickness does not necessarily parallel improvement in visual acuity, and the timing of these changes may differ [3].

Recently, the disorganization of the retinal inner layers (DRIL) has emerged as an easily identifiable imaging biomarker closely associated with visual acuity in various macular edema entities, including uveitic CME [4–6]. Large-scale, high-frequency imaging studies have shown that DRIL extent, both horizontal and vertical, as well as foveal DRIL presence, correlates strongly with worse visual acuity and with other OCT abnormalities such as cystic changes, ellipsoid zone (EZ) disruption, and the presence of hyperreflective foci (HRF) [6]. These findings support DRIL as a practical and reproducible prognostic marker of visual dysfunction.

In addition, outer retinal integrity, particularly disruption of the EZ or interdigitation zone, along with subretinal fluid (SRF) and cyst morphology or burden, have been reported to influence visual outcomes in uveitic CME. Clinical modeling studies have demonstrated that anatomical recovery, reflected by a reduction in central foveal thickness (CFT), often precedes visual improvement, and that baseline EZ integrity and the presence of SRF or cysts may predict treatment response [3]. Furthermore, increased baseline CFT has been linked with photoreceptor layer disruption and poorer visual prognosis, highlighting the clinical importance of outer retinal architecture [7].

Previous large-scale studies have demonstrated that OCT can reliably localize and delineate macular pathology, supporting its role as an essential diagnostic and prognostic tool for retinal specialists [8–10].

Identifying structural predictors at baseline may help clinicians stratify visual prognosis early and guide treatment intensity or timing more precisely. As current management relies largely on anatomical response rather than validated prognostic markers, establishing OCT-based predictors could support more individualized care and better counseling of patients with uveitic CME.

This study aims to investigate the association between pre-treatment OCT features, including DRIL, EZ and ELM integrity, SRF height, intraretinal cyst volume, and HRF count, and both baseline and post-treatment BCVA using univariate and multivariate analyses to identify potential prognostic markers.

## Materials and methods

This study was designed as a retrospective, cross-sectional analysis conducted at the Uvea Unit, Department of Ophthalmology, Ondokuz Mayis University Faculty of Medicine. The study was approved by the Ondokuz Mayis University Clinical Research Ethics Committee *(Approval No: 2022/399*,* Date: October 1*,* 2022)* and adhered to the principles of the Declaration of Helsinki.

Patients diagnosed with non-infectious uveitis who developed CME during follow-up and showed anatomical resolution of the edema after treatment between January 2016 and June 2022 were included in the study. Inclusion criteria were a diagnosis of non-infectious uveitis with CME detected during follow-up and the availability of spectral-domain OCT (SD-OCT) images suitable for quantitative analysis. Eligible eyes were required to demonstrate a CFT greater than 300 μm at any time during follow-up, accompanied by intraretinal cysts and/or subretinal fluid on OCT. Anatomical resolution after treatment was required and was defined as a reduction of at least 20% in CFT together with complete resolution of intraretinal cysts and subretinal fluid on follow-up OCT examinations.

Exclusion criteria were systemic diseases unrelated to the etiology of uveitis and ocular conditions that could affect macular morphology or visual acuity. Eyes with ocular media opacities that interfered with adequate fundus visualization or OCT interpretation, including dense corneal scarring, significant cataract (≥ grade 2 according to the LOCS III classification), posterior capsule opacification, or pronounced vitreous haze, were excluded. Eyes with macular edema related to other retinal diseases, such as diabetic retinopathy or neovascular age-related macular degeneration, were not included. OCT scans with insufficient image quality, including poor signal strength, motion artifacts, or segmentation errors that prevented reliable retinal layer assessment, were also excluded from the analysis.

**Fig. 1:**
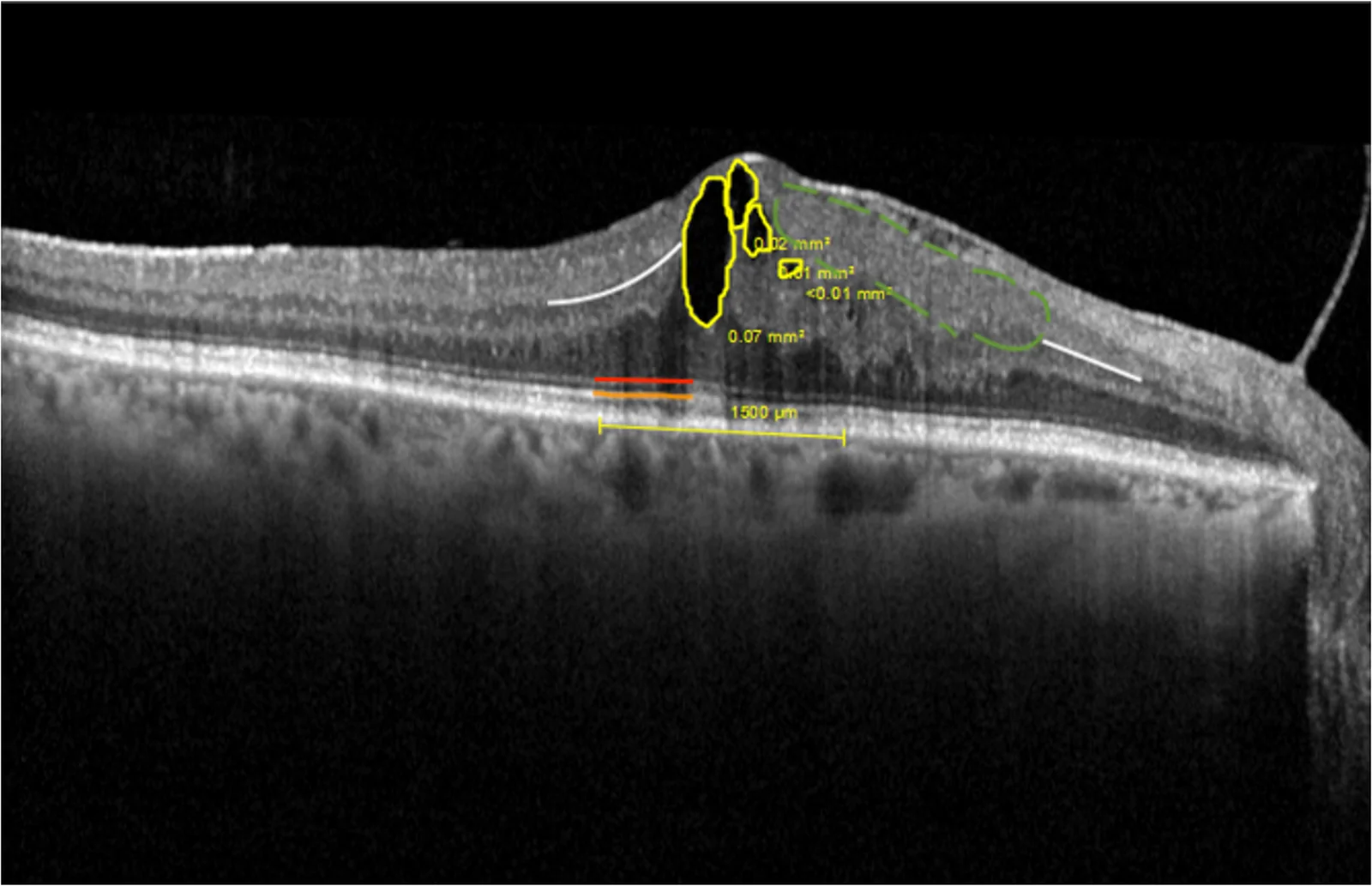
Representative spectral-domain OCT B-scan illustrating the structural biomarkers evaluated Intraretinal cysts are outlined in yellow. The orange line marks the ellipsoid zone (EZ), and the red line marks the external limiting membrane (ELM), demonstrating outer retinal integrity. Disorganization of the retinal inner layers (DRIL) is identified within the inner retinal layers, and segments exhibiting DRIL are delineated by green dashed lines. The yellow horizontal reference line indicates a central subfoveal distance of 1,500 μm and serves as a spatial scale reference

Demographic and clinical data, including age, sex, anatomical type of uveitis (intermediate, posterior, or panuveitis) and duration, BCVA, intraocular pressure, and fundus examination findings, were obtained from patient records. Spectral-domain optical coherence tomography (SD-OCT; Spectralis, Heidelberg Engineering, Heidelberg, Germany) images routinely acquired in the clinic were reviewed. All scans covered a 30 × 20-degree macular area using the GFAST mode, consisting of 25 horizontal B-scans spaced 240 μm apart. Baseline and post-treatment OCT scans were analyzed for several OCT biomarkers, including CFT, SRF height, presence and total volume of IRC, integrity of the EZ and ELM, HRF, and DRIL. All measurements were performed within the central 1500 μm macular area centered on the fovea. CFT was defined as the distance between the internal limiting membrane and the retinal pigment epithelium at the foveal center, SRF height as the maximum vertical distance between the neurosensory retina and the retinal pigment epithelium, and EZ/ELM integrity was evaluated as intact or disrupted. HRF were defined as small punctate hyperreflective lesions within the retina, and DRIL as the inability to distinguish the boundaries between the ganglion cell–inner plexiform layer complex, inner nuclear layer, and outer plexiform layer on OCT B-scans (Fig. 1).

All OCT measurements were performed using the Heidelberg Spectralis SD-OCT analysis software. Central foveal thickness was obtained using the automated measurement provided by the OCT device. Subretinal fluid height and IRC parameters, including cyst count and total cyst volume, were determined by manually delineating the relevant structures on OCT B-scans, after which the measurements were automatically calculated by the OCT device software. Qualitative OCT biomarkers, including EZ and ELM integrity and the presence of DRIL, were evaluated by an experienced ophthalmologist who was masked to the patients’ clinical data and other investigations. Since only one evaluator conducted the image assessment, intraclass correlation coefficient (ICC) analysis was not applicable. As the study was retrospective, no randomization procedures were applied.

All statistical analyses were performed using SPSS Statistics for Windows, version 22.0 (IBM Corp., Armonk, NY, USA). The normality of data distribution was assessed using the Kolmogorov–Smirnov test. As the data were not normally distributed, non-parametric tests were applied. Descriptive statistics, including mean, median, standard deviation, and frequency distribution, were provided. The Mann–Whitney U test was used for comparisons between independent groups, and Spearman’s correlation analysis was employed to examine relationships between continuous variables. Before multivariate regression analysis, multicollinearity among OCT biomarkers was assessed using variance inflation factor (VIF) and tolerance statistics. To identify independent predictors of final BCVA, multivariate linear regression analysis was performed. A *p*-value of less than 0.05 was considered statistically significant.

## Results

A total of 84 eyes from 66 patients diagnosed with CME secondary to non-infectious uveitis were included in the study. The mean age of the patients was 38.9 ± 19.2 years, and 54.5% were female. The mean treatment duration was 17.6 ± 20.2 weeks. The etiologic distribution among the 66 patients included intermediate uveitis (*n* = 11) and idiopathic posterior uveitis (*n* = 16), while panuveitis etiologies included Behçet disease (*n* = 14), idiopathic panuveitis (*n* = 14), Vogt–Koyanagi–Harada disease (*n* = 4), sarcoidosis (*n* = 3), sarcoid-like uveitis (*n* = 1), tuberculosis-associated uveitis (*n* = 1), and sympathetic ophthalmia (*n* = 2).

Before treatment, mean BCVA was 0.70 ± 0.43 logMAR (approximately 20/100), which improved to 0.35 ± 0.38 logMAR (approximately 20/45), corresponding to an average gain of roughly 17 ETDRS letters (*p* < 0.001). Similarly, the mean CFT decreased from 538.35 ± 216.24 μm at baseline to 252.63 ± 62.07 μm following treatment (*p* < 0.001).

According to OCT, the EZ and ELM integrity were disrupted in 38 eyes (45.2%). DRIL was detected in 21 eyes (25.0%), SRF in 58 eyes (69.0%), IRC in 71 eyes (84.5%), and HRF in 78 eyes (92.8%). The mean SRF height was 151.6 ± 129.7 μm, total IRC volume was 0.21 ± 0.17 mm³, and the mean number of HRF was 16.94 ± 14.05.

In univariate linear regression analysis, baseline BCVA showed significant associations with CFT (*p* < 0.001), DRIL presence (*p* = 0.021), and SRF height (*p* = 0.002). These associations were further tested in the multivariate model to evaluate whether they independently predicted visual outcome. In multivariate regression analysis, CFT (*p* = 0.038) and SRF height (*p* = 0.049) remained independently associated with baseline BCVA (Table 1).

**Table 1:** Univariate and multivariate linear regression analyses of baseline OCT parameters associated with baseline BCVA (logMAR)

Model 1	Unstandardized Coefficients (B)		Standardized Coefficients (Beta)	t	*p* value	95.0% CI for B (Lower - Upper)	
	B	Std. Error	Beta			Lower	Upper
(Constant)	0.932	0.152		6.140	0.000	0.630	1.234
EZ Disrupt	-0.149	0.093	-0.174	-1.599	0.114	-0.335	0.036
DRIL	-0.249	0.106	-0.252	-2.361	0.021	-0.459	-0.039
SRF	0.017	0.102	0.019	0.168	0.867	-0.186	0.220
SRF Height	0.001	0.000	0.406	3.323	0.002	0.001	0.002
Total IRC Count	0.006	0.013	0.052	0.436	0.664	-0.021	0.032
Total IRC Volume	0.406	0.302	0.160	1.346	0.183	-0.196	1.008
HRF	0.004	0.003	0.119	1.086	0.281	-0.003	0.010
Baseline CFT	0.001	0.000	0.388	3.814	0.000	0.000	0.001

Model 2	Unstandardized Coefficients (B)		Standardized Coefficients (Beta)	t	*p* value	95.0% CI for B (Lower - Upper)	
	B	Std. Error	Beta			Lower	Upper
(Constant)	0.070	0.396		0.177	0.861	-0.725	0.865
Baseline CFT	0.001	0.000	0.318	2.124	0.038	0.000	0.001
Age	0.003	0.003	0.145	1.130	0.263	-0.002	0.009
Sex	0.007	0.105	0.008	0.065	0.948	-0.203	0.217
DRIL	0.020	0.130	0.022	0.155	0.877	-0.242	0.282
SRF Height	0.001	0.000	0.297	2.013	0.049	0.000	0.002

Model-1: Univariate Linear Regression Analyses; Model-2: Multivariate Linear Regression Analyses;

*ELM* External Limiting Membrane, *EZ* Elipsoid Zone, *DRIL* Disorganized Retinal Inner Layer, *SRF* Subretinal Fluid, *IRC* Intraretinal Cyst, *HRF* Hyperreflective Foci

Regarding final BCVA, univariate analysis revealed significant associations with EZ disruption (*p* = 0.007), ELM disruption (*p* = 0.007), total intraretinal cyst volume (*p* = 0.010), and baseline CFT (*p* < 0.001). In the multivariate regression model, only baseline CFT (*p* = 0.006) remained an independent predictor of final BCVA (Table 2).

**Table 2:** Univariate and multivariate linear regression analyses of baseline OCT parameters associated with final BCVA (logMAR)

Model 1	Unstandardized Coefficients (B)		Standardized Coefficients (Beta)	t	*p* value	95.0% CI for B (Lower - Upper)	
	B	Std. Error	Beta			Lower	Upper
Constant	0.706	0.133		5.303	0.000	0.441	0.972
ELM Disrupt	-0.225	0.082	-0.290	-2.745	0.007	-0.388	-0.062
EZ Disrupt	-0.225	0.082	-0.290	-2.745	0.007	-0.388	-0.062
DRIL	-0.191	0.096	-0.214	-1.988	0.050	-0.382	0.000
SRF	0.114	0.091	0.136	1.246	0.216	-0.068	0.296
SRF Height	0.001	0.000	0.207	1.586	0.118	0.000	0.001
Total IRC Count	0.000	0.012	0.003	0.029	0.977	-0.024	0.024
Total IRC Volume	0.700	0.266	0.302	2.632	0.010	0.169	1.230
HRF	0.000	0.003	-0.011	-0.096	0.924	-0.006	0.006
Baseline CFT	0.001	0.000	0.429	4.303	0.000	0.000	0.001

Model 2	Unstandardized Coefficients (B)		Standardized Coefficients (Beta)	t	*p* value	95.0% CI for B (Lower - Upper)	
	B	Std. Error	Beta			Lower	Upper
Constant	-0.213	0.349		-0.611	0.543	-0.911	0.484
Sex	-0.094	0.091	-0.118	-1.037	0.303	-0.276	0.087
DRIL	0.037	0.119	0.042	0.314	0.755	-0.200	0.275
Total IRC Volume	0.420	0.338	0.181	1.242	0.219	-0.256	1.096
ELM Disrupt	-0.022	0.116	-0.028	-0.193	0.848	-0.255	0.210
Baseline CFT	0.001	0.000	0.345	2.263	0.006	0.000	0.001
Age	0.007	0.002	0.359	3.097	0.053	0.003	0.012

Model-1: Univariate Linear Regression Analyses; Model-2: Multivariate Linear Regression Analyses

*ELM* External Limiting Membrane, *EZ* Elipsoid Zone, *DRIL* Disorganized Retinal Inner Layer, *SRF* Subretinal Fluid, *IRC* Intraretinal Cyst, *HRF* Hyperreflective Foci

Collinearity diagnostics demonstrated no significant multicollinearity among the variables included in the final regression models (all VIF values < 5 and tolerance values > 0.2).

## Discussion

In this study, we evaluated the relationship between baseline OCT findings and both baseline and post-treatment BCVA in eyes with CME secondary to non-infectious uveitis. Our results demonstrated that greater baseline CFT was significantly associated with poorer BCVA both at baseline and after treatment. Although EZ and ELM disruption, DRIL presence, higher IRC volume, and increased SRF height demonstrated associations with visual outcomes in univariate analysis, these parameters did not remain significant after adjustment in the multivariate model. Therefore, they should not be interpreted as independent predictors, but rather as secondary structural features that may reflect the degree of retinal involvement. Because multiple OCT abnormalities frequently coexisted in the same eye, attributing changes in BCVA to a single structural biomarker may be challenging. Therefore, the relationship between each OCT biomarker and BCVA was first evaluated using univariate analyses. Subsequently, multivariate regression analysis was performed to determine the independent contribution of each biomarker to BCVA while adjusting for the presence of other OCT features.

The presence of DRIL has been identified as a major microstructural disturbance that limits visual recovery across different macular pathologies, particularly in eyes with diabetic macular edema [11, 12], retinal vein occlusion [13, 14], and idiopathic epiretinal membrane [15]. This observation is consistent with previous studies suggesting that DRIL serves as a reliable structural biomarker of visual function. Grewal et al. reported a strong correlation between DRIL extent and visual acuity, indicating that this parameter reflects foveal microstructural disruption [6]. Similarly, Liu et al. showed that eyes with DRIL exhibited significantly lower visual acuity both at baseline and during follow-up compared with eyes without DRIL, and that the extent of baseline DRIL was significantly associated with final BCVA [16]. In our study, the presence of DRIL was associated with lower baseline BCVA, consistent with previous reports. In contrast, although DRIL was associated with baseline BCVA in our cohort, it did not remain independently associated with post-treatment BCVA after adjustment for other OCT biomarkers. These findings suggest that DRIL may not only indicate existing anatomical damage but also represent the potential for visual improvement. However, in our study, DRIL did not remain an independent predictor of post-treatment BCVA after adjustment in the multivariate model. Similar findings have been reported in diabetic macular edema, where Zur et al. demonstrated that eyes without DRIL at baseline achieved significantly better visual and anatomical outcomes after dexamethasone treatment, further supporting DRIL as a clinically meaningful prognostic biomarker across macular edema etiologies [9].

Structural integrity of the outer retina, particularly the EZ and ELM, is another key determinant of visual prognosis [17]. In our cohort, eyes with EZ and ELM disruption exhibited significantly poorer final visual acuity. This result aligns with previous evidence emphasizing that preservation of the EZ, corresponding to photoreceptor outer segments, is essential for maintaining visual function. Ciulla et al. reported that EZ integrity in noninfectious uveitic eyes predicted treatment response, with anatomical improvement preceding functional recovery [3]. Similarly, Alvarez-Guzman et al. demonstrated that increased central macular thickness was associated with outer retinal disruption, reflecting photoreceptor damage and a higher risk of permanent visual loss highlighting the importance of photoreceptor integrity for visual prognosis [7]. Therefore, evaluating EZ and ELM integrity may provide valuable information for predicting treatment response and long-term visual outcomes. Because photoreceptor integrity is essential for functional recovery after resolution of macular edema, disruption of the EZ or ELM is generally considered an indicator of limited visual improvement following treatment. Previous studies have also demonstrated that disruption of outer retinal microstructure is closely associated with visual function in uveitic macular edema. Tortorella et al. reported that defects of the photoreceptor layers, including disruption of the ellipsoid zone and the interdigitation zone, were significantly correlated with worse BCVA in multivariate analysis, highlighting the importance of outer retinal integrity for visual prognosis in these patients [18]. In our study, although EZ and ELM disruption were associated with poorer post-treatment BCVA in univariate analyses, they did not remain independent predictors after multivariate adjustment. This suggests that the apparent association between outer retinal disruption and visual outcome may partly reflect the coexistence of other structural OCT abnormalities, such as increased retinal thickness or intraretinal cystic changes. In agreement with our findings, Zur et al. further highlighted that preserved external retinal integrity, including intact IS–OS continuity and the presence of subretinal fluid, served as favorable prognostic indicators in diabetic macular edema treated with dexamethasone implants, reinforcing the relevance of these structural OCT signatures in predicting functional potential [10].

In our study, increased CFT was found to be associated with lower BCVA both at baseline and post-treatment. This finding is consistent with prior research demonstrating that higher baseline CFT values can predict outer retinal layer disruption and worse visual prognosis in uveitic CME [19, 20]. Previous studies showed that eyes with CFT > 337 μm had a significantly greater likelihood of ellipsoid (EZ) and interdigitation zone (IZ) loss, which markedly increased the risk of severe visual impairment [7]. Ciulla et al. similarly observed that eyes with preserved EZ integrity achieved greater post-treatment visual improvement, whereas those with thicker foveae exhibited limited recovery [3].

Moreover increased total IRC volume was found to be significantly associated with lower final BCVA. Enlargement of cystoid spaces within the inner retina may disrupt the retinal microarchitecture, impair synaptic connectivity between photoreceptors and bipolar cells, and ultimately limit visual recovery. Previous studies reported that cyst volume inversely correlated with visual acuity and paralleled the extent of DRIL and outer retinal disruption [6]. Recent studies have also demonstrated that automated deep learning–based algorithms can provide reliable intraretinal cyst/fluid segmentation and volumetric quantification with high concordance to expert grading in various retinal diseases [21, 22]. Nevertheless, because IRC volume measurements in the present study were performed manually/semi-manually, these findings should still be interpreted with appropriate caution.

The number of HRF was not significantly correlated with final visual acuity in our study, these structures are considered relevant biomarkers reflecting inflammatory activity and microstructural disorganization in uveitic macular edema. Berasategui et al. prospectively investigated HRF behavior in noninfectious uveitic macular edema and observed a significant reduction in HRF number over 12 months, along with a shift from the outer to the inner retinal layers following treatment [23]. This finding suggests that HRFs are dynamic structures that regress as inflammation subsides. In addition, studies by Chen and Kang demonstrated that HRF density correlates with photoreceptor integrity and visual function, and may represent activated microglial cells or extravasated lipoprotein deposits [24, 25]. Therefore, even though HRF count was not an independent prognostic factor in our series, its presence may still provide complementary information regarding the severity and duration of retinal inflammation in noninfectious uveitis. Furthermore, previous studies have demonstrated that automated deep learning–based HRF quantification methods in retinal diseases such as diabetic macular edema, retinal vein occlusion, and central serous chorioretinopathy showed good agreement with expert ophthalmologist assessments [26, 27]. Nevertheless, because HRF evaluation was performed manually in the present study, the results related to this parameter should still be interpreted with caution.

Previous studies have reported that SRF may carry prognostic value in uveitic macular edema. Lehpamer et al., in a retrospective study involving 101 eyes with uveitic macular edema, found that the presence of SRF was linked to a nearly sixfold higher likelihood of edema regression and better visual outcomes after treatment [28]. Similarly, Cicinelli et al. suggested that SRF in noninfectious uveitic macular edema tends to occur in eyes with shorter disease duration and may indicate preserved Müller cell and foveal cone integrity, often associated with faster anatomical improvement after intravitreal dexamethasone therapy [2]. These findings suggest that SRF may represent an important morphological marker associated with visual prognosis in uveitic CME and, in some cases, may reflect a reversible inflammatory phase of the disease. In our study, increased SRF height was significantly associated with lower baseline BCVA in both univariate and multivariate analyses. Additionally, the importance of OCT-guided evaluation in macular edema management has been demonstrated in multicenter real-world studies. Iglicki et al. reported that longitudinal OCT-based monitoring of retinal thickness correlated strongly with visual improvement following dexamethasone implantation, particularly in treatment-naïve patients, supporting the role of OCT-derived parameters in guiding therapeutic strategies [8].

### Strengths and limitations

The main strength of this study lies in the comprehensive evaluation of multiple baseline OCT biomarkers in noninfectious uveitic CME and the analysis of their associations with both baseline and post-treatment BCVA. By simultaneously assessing both inner and outer retinal integrity, this study provides a more complete understanding of the structure–function relationship in uveitic macular edema.

However, the retrospective design and relatively small sample size represent important limitations. Variability in treatment protocols may act as a potential confounding factor. Although manual OCT measurements may carry a risk of observer-related bias, the fact that all measurements were performed by a single examiner enhanced standardization and ensured measurement consistency across evaluations. HRF number and intraretinal cyst parameters were assessed on OCT B-scans (line scans); HRF were manually counted, whereas intraretinal cyst parameters were obtained by manually delineating the relevant structures, after which the measurements were automatically calculated by the Heidelberg Spectralis SD-OCT device software. In addition, although recent studies have demonstrated that automated deep learning–based algorithms may provide reliable HRF and intraretinal cyst/fluid quantification with high concordance to expert grading in various retinal diseases, HRF and IRC volume assessments in the present study were performed using manual/semi-manual methods. Therefore, the findings related to these parameters should be interpreted with appropriate caution.

## Conclusion

This study indicates that among baseline OCT parameters evaluated in eyes with cystoid macular edema secondary to noninfectious uveitis, baseline CFT was the only independent predictor of post-treatment visual outcome in multivariate analysis. Several other OCT features, including DRIL, SRF height, EZ/ELM disruption, and IRC volume, showed associations with visual acuity in univariate analysis but did not remain significant after adjustment for covariates. These findings suggest that while multiple structural abnormalities reflect the burden of retinal involvement, baseline CFT may serve as the most reliable and practical prognostic metric in routine clinical assessment. Further prospective and multicenter studies are needed to validate these associations and refine predictive modeling in uveitic macular edema.

## Data Availability

The datasets generated during and/or analyzed during the current study are available from the corresponding author on reasonable request.
